# Raman Spectral Signatures of Human Neutrophils: A Single‐Cell Approach

**DOI:** 10.1002/jbio.70251

**Published:** 2026-03-10

**Authors:** Lenka Vaňková, Jiří Bufka, Pavla Šigutová, Monika Holubová, Věra Křížková

**Affiliations:** ^1^ Faculty of Medicine in Pilsen Charles University in Prague Pilsen Czech Republic; ^2^ Motol University Hospital Prague Czech Republic; ^3^ Institute of Organic Chemistry and Biochemistry, Czech Academy of Sciences Prague Czech Republic; ^4^ University Hospital in Pilsen Pilsen Czech Republic; ^5^ Biomedical Center, Faculty of Medicine in Pilsen Charles University Pilsen Czech Republic

**Keywords:** hematological smear, molecular fingerprinting, neutrophils, neutrophils spectra, Raman microscopy

## Abstract

**Introduction:**

With their noninvasive capabilities for molecular‐level analysis of biological tissues and cells, Raman spectroscopy and microscopy have become important tools in medicine and medical diagnostics.

**Methods:**

In this work, we describe a reliable and repeatable technique for Raman spectroscopic profiling of human neutrophils in peripheral blood smears. 1200 single‐cell spectra from 12 healthy donors were obtained using Raman microscopy, and the results showed consistent spectral features of key biomolecules such as proteins, nucleic acids, carotenoids, and cytochrome c.

**Results:**

We used t‐distributed stochastic neighbor embedding and principal component analysis to assess interindividual variability; both techniques showed minimal spectral divergence between donors and high reproducibility. This study lays the methodological groundwork for upcoming Raman microscopy applications enabling non‐invasive assessment of immune cell states in health and disease.

**Conclusion:**

Our results demonstrate the potential of Raman microscopy in clinical diagnostics, specifically for tracking the metabolic states of cells.

## Graphical abstract

In this study, a standardized Raman microscopy protocol was established for single‐cell analysis of human neutrophils directly from routine hematological smears. Highly reproducible molecular fingerprints with low interindividual variability were obtained. This methodology provides a robust reference framework for future biophotonic studies of neutrophil states in health and disease.

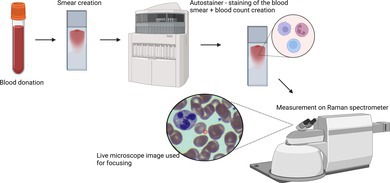

## 1 Introduction

### 1.1 Raman Spectroscopy

Raman spectroscopy (RS) is a nondestructive analytical technique that leverages the interaction of electromagnetic radiation with the molecules in a sample. The resulting spectra provide detailed information about molecular geometry, vibrational modes, and the chemical groups and molecular symmetry present in the sample [1]. This method has been used to identify various cellular components, such as carbohydrates, lipids, amino acids, nucleic acids, and carotenoids, with each molecule exhibiting a unique spectral “fingerprint” [2, 3, 4].

The sample under examination is illuminated by laser light, which undergoes inelastic scattering. During this process, a photon transfers a portion of its energy to the sample molecules, causing them to transition to excited vibrational states. The Raman spectrum reflects the intensity of the scattered radiation and the shift in wavelength, known as the “Raman shift.” This can be measured using a Raman spectrometer or, with spatial resolution, a Raman microscope [5]. The bands observed in the Raman spectrum correspond to specific molecular vibrations [6].

In recent years, RS and microscopy have shown great promise in the biomedical field, particularly for imaging the chemical composition of cells and tissues and analyzing cellular metabolism [3, 7]. Due to its noninvasive nature, flexibility, and rapid measurement capabilities, RS holds potential as a diagnostic tool for studying molecular processes in medicine, including in the hematological field.

### 1.2 Neutrophils and Their Molecular Properties

As an essential component of the immune system, neutrophils exhibit unique chemical characteristics that support their vital role in defense. They are easily identifiable by their polymorphonuclear structure, which typically consists of 1–5 lobes connected by thin strands of chromatin. This distinctive shape is closely linked to their function. Neutrophils contain three types of granules: azurophilic (primary), specific (secondary), and tertiary. The azurophilic granules are rich in enzymes such as neutrophil elastase, myeloperoxidase, and defensins. Specific granules contain enzymes including alkaline phosphatase, lysozyme, lactoferrin, and various proteases. Tertiary granules primarily contain gelatinase and collagenase [8].

Neutrophils express a range of cell surface receptors that enable them to detect and respond to inflammatory signals. These include chemokine receptors, such as the IL‐8 receptor (CXCL8), which directs neutrophil migration to infection sites. They also express complement receptors, including C3a and C5a, which facilitate the recognition and phagocytosis of opsonized pathogens, boosting their ability to eliminate invaders. Additionally, neutrophils possess pattern recognition receptors, including toll‐like receptors that recognize pathogen‐associated molecular patterns and trigger immune responses, enhancing the neutrophils' defensive capabilities [9]. To facilitate their movement during the inflammatory response, neutrophils utilize adhesion molecules, such as integrins (e.g., CD11b/CD18) and selectins, to adhere to the endothelium. This adhesion is crucial for their effective migration from the bloodstream to surrounding tissues, where they can combat infections. Upon activation, neutrophils exhibit changes in surface expression of various markers, such as the upregulation of CD66b, associated with granule release, and CD11b, which enhances their ability to adhere to endothelial cells and phagocytose pathogens [10].

Neutrophils are among the first responders to infections, playing a key role in recruiting other immune cells to the affected areas. They can carry out effector functions rapidly, often without needing cues from other cells. Neutrophils comprise 60%–70% of leukocytes in peripheral blood, with approximately 93% residing in the bone marrow and only 7% circulating in the bloodstream. These proportions can be altered by bacterial products and inflammatory cytokines [11].

As highly phagocytic cells, neutrophils are capable of engulfing pathogens. After ingestion, they release enzymes from their granules into phagosomes to eliminate the bacteria. A hallmark feature of activated neutrophils is the production of reactive oxygen species via the NADPH oxidase complex. This oxidative burst is essential for killing engulfed pathogens. To prevent oxidative damage, neutrophils are equipped with antioxidant systems, including enzymes such as catalase and superoxide dismutase, which help maintain redox balance within the cell [8]. Neutrophils also secrete various cytokines that amplify the inflammatory response and recruit additional immune cells to the site of infection. Recent studies have shown that lipid droplets in neutrophils are associated with specific biological processes, and their composition can change depending on the level of neutrophil activation and pathological conditions [12].

Another important functional mechanism of neutrophils is the formation of neutrophil extracellular traps (NETs) [13]. NETs consist of a network of cell‐free DNA, histones, and granule‐derived proteins, including microbicidal enzymes. While the primary function of NETs is to trap pathogens, their formation can also trigger inflammatory and immune responses. In some cases, pathogen‐induced NETs may lead to uncontrolled inflammation, contributing to an exaggerated systemic inflammatory response [14, 15].

### 1.3 Raman Spectroscopy in Neutrophil Research

Hematology and research involving blood and its components have long been key areas for the application of RS. This widespread use is due in part to the ease of obtaining blood samples, as well as the valuable insights blood analysis provides into an organism's overall physiology and pathology. Beyond the whole blood, RS has also been extensively used in studies of plasma and serum, as well as in the investigation of specific blood components.

The earliest studies that applied RS to neutrophils primarily focused on differentiating the nucleus and cytoplasm of leukocytes in general [16]. Subsequent studies expanded on this by describing methods for distinguishing between different types of leukocytes using RS [17]. However, research focused exclusively on neutrophils remains relatively limited. Nonetheless, RS has proven valuable in examining neutrophil activation states, responses to pathogens, and overall cellular mechanisms.

RS has been successfully used to differentiate between bacterial and fungal infections by analyzing neutrophils. In one study, neutrophils isolated from human blood were stimulated with various pathogens, including *Staphylococcus aureus* , *Escherichia coli* , and the fungus *Candida albicans* . This technique facilitated the identification of phagocytized pathogens and allowed for the differentiation of infected from noninfected cells, achieving an impressive accuracy rate of 90%. Furthermore, RS was able to distinguish between bacterial and fungal infections with an accuracy of 92% [18]. Neutrophil responses to viral exposure have also been studied using RS, shedding light on their in vitro activation and the cellular changes associated with viral infection [19].

RS has also been employed to study how neutrophils interact with exogenous substances, such as multi‐walled carbon nanotubes. This approach enhances our understanding of neutrophil involvement in immune responses by providing insights into the activation and functional reactions of neutrophils to various stimuli [20].

Additionally, Raman microscopy has proven effective in differentiating NETs from other forms of cell death, such as necrosis. By analyzing molecular composition through specific vibrational bonds, researchers have identified unique spectral signatures for NETs, distinguishing them from necrotic cells. This differentiation is important, as traditional techniques such as immunofluorescence often struggle to accurately differentiate between these cell types. The use of machine learning techniques to analyze Raman spectra has further enhanced the classification of NETs, revealing differences in molecular composition based on specific stimuli that induce their formation [21].

Although neutrophils and their characteristics have been examined using Raman spectroscopy in several studies, the experimental setups used are inconsistent. Important methodological elements such as cell isolation protocols, fixation techniques, excitation wavelengths, objective magnification, laser power settings, and others differ greatly from study to study and are not always adequately explained. This variability restricts the reproducibility of Raman‐based immune cell profiling and makes it more difficult to compare results. Considering this, transparent and standardized protocols are required to enable consistent spectrum acquisition and interpretation.

### 1.4 Objectives of the Present Study

Our goal was to obtain representative neutrophil samples from healthy individuals and establish a standardized methodology for their processing. We also aimed to investigate interindividual variability and analyze the relative intensities and positions of specific Raman bands, with the objective of assigning them to biologically relevant molecules. We present a detailed protocol for neutrophil sample preparation and demonstrate the application of Raman spectroscopy to detect normal neutrophils and characterize their subcellular molecular composition.

## 2 Materials and Methods

### 2.1 Ethics Statement

Informed consent for the use of biological material for research purposes was obtained in accordance with the protocol approved by the Ethics Committee of the University Hospital Pilsen and the Faculty of Medicine in Pilsen, Charles University (Reference Number 458/22). Fresh blood samples were processed and provided by the Department of Clinical Biochemistry and Hematology, University Hospital Pilsen, following institutional guidelines.

### 2.2 Sample Collection and Processing

**FIGURE 1:**
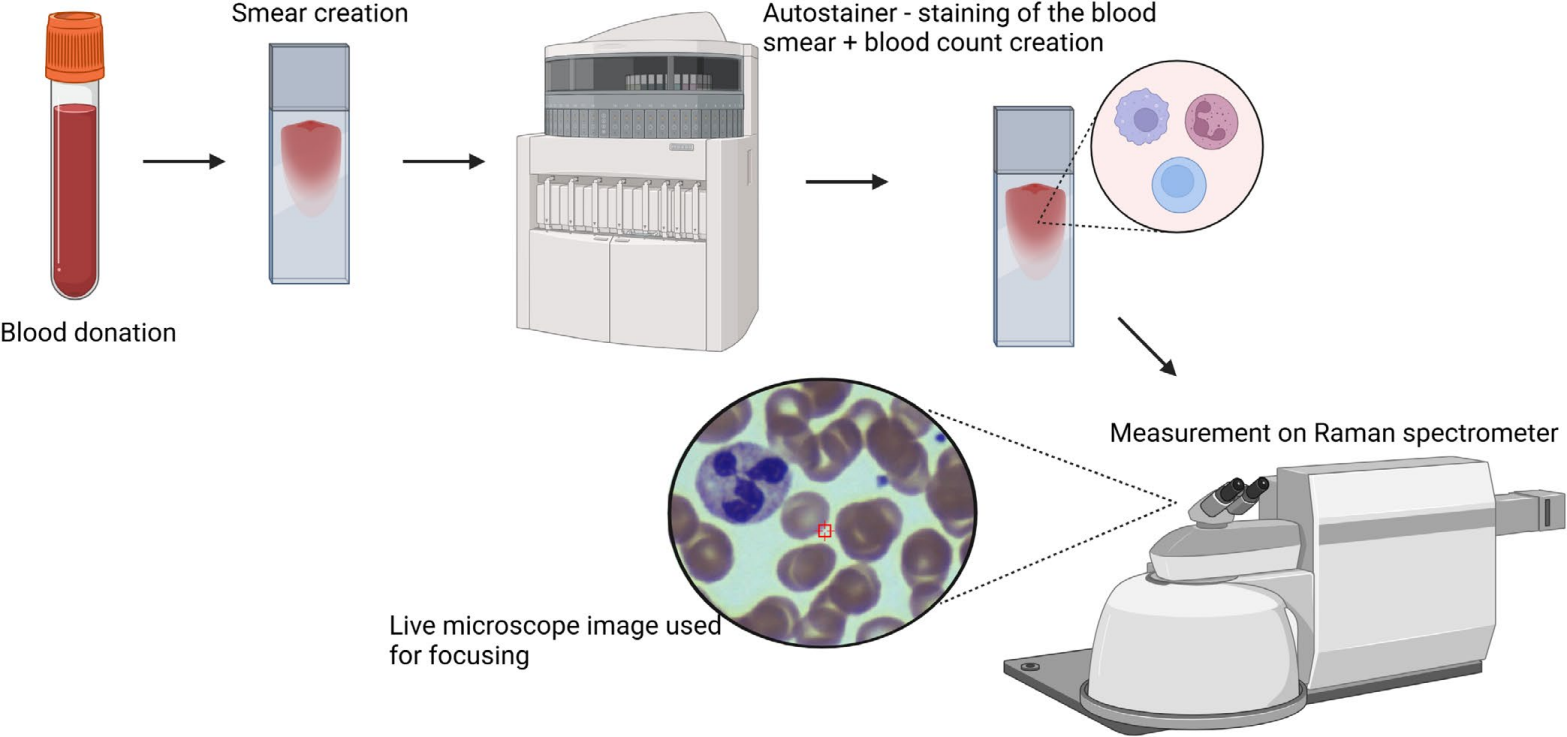
Schematic representation of the neutrophil processing protocol. The figure was created using BioRender.com.

Blood smears were prepared from peripheral blood samples collected from 12 healthy donors. The donors were confirmed to be in good health and free from acute illness and were not taking any medications. A standard blood count was also performed on the samples to confirm the absence of inflammation and other acute conditions. Blood was drawn into EDTA‐anticoagulated tubes and immediately applied to clean quartz slides. The established protocol for blood smear preparation was followed. The smears were then stained using the standard May‐Grünwald‐Giemsa staining method, which includes fixation with methanol. Blood smear preparation and staining were automated using the UniCel DxH Slidemaker Stainer (Beckman Coulter, USA) with glass slides provided by SYSMEX. The staining procedure was carried out according to the laboratory's established protocols. A simplified schematic of the neutrophil processing protocol is shown in Figure 1.

### 2.3 Raman Spectroscopy Procedure

The prepared blood smears were transferred to the laboratory on the same day and analyzed using a Raman spectrometer. Single‐cell Raman spectra were recorded using a Raman microscope (Olympus, Japan) coupled with a Raman spectrometer (DXR1, Thermo Scientific, USA), employing a 100× immersion objective (Olympus, Japan) and a diode laser with a wavelength of 532 nm. A 532 nm excitation wavelength was selected due to its high Raman scattering efficiency and its suitability for detecting intracellular chromophores, particularly carotenoids and cytochrome c. Laser power at the sample was maintained at low levels (≤ 0.2 mW) and remained constant during each individual spectral acquisition. Laser power was adjusted in relation to the acquisition time to maintain a comparable signal‐to‐noise ratio while minimizing laser‐induced effects, with shorter acquisition times combined with higher laser power and longer acquisition times performed at lower power. Under the laser powers used, no visible cellular damage was observed during measurement. The objective lens in the microscope also collected backscattered light from the sample, which was then filtered through a holographic notch filter, directed to the spectrometer, and detected by a charge‐coupled device detector. Cells were selected for measurement using the microscope's standard visual mode, and at least 100 cells were measured per sample. For each neutrophil, a single Raman spectrum was acquired by focusing the laser on the nuclear region. One spectrum per cell was recorded to avoid repeated laser exposure and potential photodamage.

Only neutrophils with standard morphology and 2–3 nuclear segments as assessed on May‐Grünwald and Giemsa‐Romanovski smears were selected to ensure uniform cell age. All measurements were completed within 24 h of blood collection. Previous experiments confirmed the stability of the samples over this time period using our processing method.

### 2.4 Raman Spectral Acquisition and Data Analysis

A total of 1200 cells were analyzed, with spectra acquired from neutrophils of 12 different healthy donors. In our study, the laser was focused on the nuclear region, which yielded the highest Raman signal‐to‐noise ratio. The nucleus was identified as the optimal region for obtaining the best signal‐to‐noise ratio in the Raman spectra. However, considering the scattering volume in the experiment, the spectra also contained average information from the cytosol and cell membrane. Raman spectra were processed using Omnic software (Thermo Scientific, USA). Further analyses were carried out in a Google Colaboratory environment with open‐source scientific libraries in Python First, asymmetric least squares baseline correction was used to adjust the raw spectra for background fluorescence. All spectra were interpolated onto a common Raman shift axis (1000 uniformly spaced points) and normalized to unit range (0–1) using min‐max scaling to enable inter‐sample comparability. Additionally, to account for any potential interference from chemicals used in blood smear preparation (such as stains and methanol) and immersion oil, their spectra were measured separately, and their contributions were excluded from the final neutrophil spectra. Because of the small sample size, inter‐individual variability was evaluated using principal component analysis (PCA) and t‐distributed stochastic neighbor embedding (t‐SNE) using a low perplexity value to emphasize local relationships, which is suitable for small sample sizes. Characteristic Raman bands were attributed to common cellular biomolecules present in neutrophils, and peak detection was carried out on the mean normalized spectrum using a peak prominence threshold algorithm. To assess interindividual variation in the intensities of selected Raman peaks, the coefficient of variation (CV) was calculated across the 12 donors for each of the ten most prominent spectral bands. Peak assignments were made based on existing literature.

## 3 Results

**FIGURE 2:**
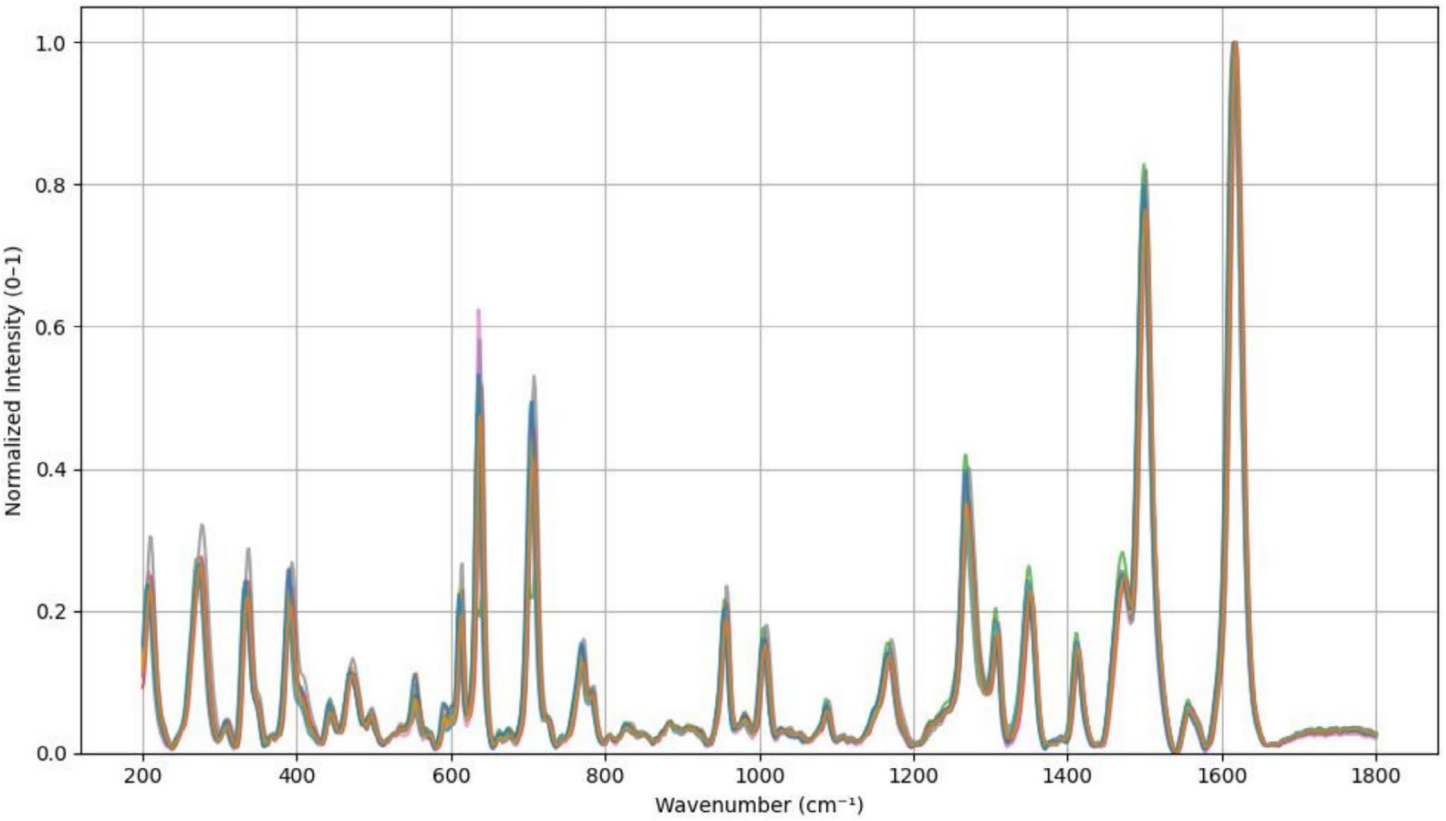
Averaged Raman spectra of neutrophils from 12 healthy donors. Each curve represents the average of 100 single‐cell spectra obtained from an individual donor. Spectra were baseline‐corrected, interpolated to a uniform Raman shift axis, and min–max normalized. Only the spectral region between 200 and 1800 cm^−1^ is shown.

We profiled neutrophils from 12 healthy donors with verified hematological classification to confirm cell morphology and health status. In Figure 2, the mean Raman spectra of cells from each donor are displayed and the spectra were baseline‐corrected, interpolated to a uniform Raman shift axis, and min–max normalized. Only the spectral region between 200 and 1800 cm^−1^ is shown. The Raman spectra were reproducible, and the same molecular features were present through the different donors. To quantify interindividual variability, we calculated the coefficient of variation (CV) for the ten most prominent Raman peaks (Table 1). CV values ranged from 19.5% to 27.2%, indicating low to moderate yet biologically acceptable interindividual variability. The most stable peaks were located at 1618, 388, and 471 cm^−1^, while the highest variability was observed at 1410 cm^−1^.

**TABLE 1:** Statistical summary of the ten prominent Raman peaks observed in neutrophils.

Raman shift (cm^−1^)	Mean intensity	Standard deviation	CV (%)
1410	1418.65	385.45	27.17
1348	2400.89	578.92	24.11
637	5132.36	1225.30	23.87
706	4615.08	1070.66	23.20
1307	1800.40	394.80	21.93
1169	1428.56	296.29	20.74
1500	8768.79	1760.65	20.08
471	1098.90	218.88	19.92
388	2249.25	444.90	19.78
1618	11437.93	2230.15	19.50

*Note:* The table includes peak position, mean intensity, standard deviation (SD), and coefficient of variation (CV) calculated across 12 donors.

**FIGURE 3:**
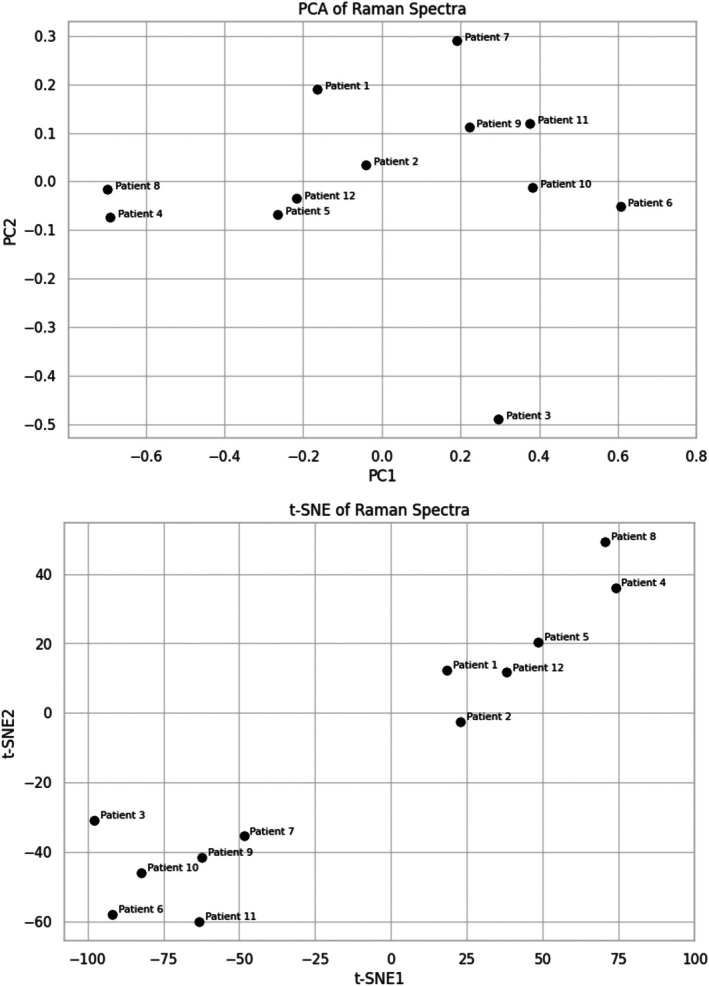
Principal component analysis (PCA) plot of Raman spectra and t‐distributed stochastic neighbor embedding (t‐SNE) plot from neutrophils of 12 donors. Each point represents the average spectrum of a single donor. The tight clustering of samples in PCA indicates low interindividual variability in the spectral features. t‐SNE plot visualizes the spectral similarity of neutrophil Raman profiles. Spectra cluster closely together, confirming high reproducibility and similarity between individual donors.

To further evaluate donor‐level similarity, we performed principal component analysis (PCA) and t‐distributed stochastic neighbor embedding (t‐SNE) on the averaged spectra. Figure 3 shows the PCA score plot based on the average spectra of all donors, revealing tight clustering with minimal spread. These findings confirm tight clustering with minimal interindividual variation. Along the same lines, the results of the t‐SNE analysis (Figure 3) point to high spectral similarity with only minimal separation among donors. These results are consistent with the idea that neutrophil Raman signatures are highly reproducible under standardized conditions.

Table 2 illustrates tentative peak assignments. We identified several distinct bands corresponding to different biomolecules, including proteins (270, 334, 388, 442, 471, 1168, 1410, 1556, and 1618 cm^−1^), nucleic acids (612, 637, 706, 770, 1307, and 1471 cm^−1^), carotenoids (955, 980, 1006, 1168, and 1500 cm^−1^), cytochrome C (1268 and 1348 cm^−1^) [22, 23, 24, 25, 26, 27, 28]. As expected due to methanol fixation, no peaks typical for lipid structures were observed.

**TABLE 2:** Assignment of selected Raman bands in neutrophil spectra to vibrations of potential biomarkers.

Substances	Wavelength (cm^−1^)
Proteins	270, 334, 388, 442, 471, 1169, 1410, 1556, and 1618
Nucleic acids	612, 637, 706, 770, 1307, and 1471
Carotenoids	955, 980, 1006, 1168, and 1500
Cytochrome C	1268 and 1348

## 4 Discussion

Raman spectroscopy has previously been applied to various types of immune cells, including lymphocytes, monocytes, and neutrophils, as described in the introduction. These techniques have been used to study cellular activation, differentiation, and pathological alterations. However, the methodologies used in different studies often varied considerably, including differences in sample preparation, excitation wavelength, laser power, and spectrum acquisition settings. In addition, the subsequent statistical analysis of Raman data is often inconsistent or poorly described. This variability limits the comparability, reproducibility, and wider applicability of Raman‐based immune profiling.

In contrast, our research presents a consistent and clearly described method for measuring neutrophils directly from standard hematological smears. Our dataset, comprising 1200 single‐cell spectra from 12 healthy donors, provides a good basis for determining the basic molecular features of physiological neutrophils. Spectral consistency was high, as demonstrated by tight clustering in PCA and t‐SNE and low coefficients of variation. The slight differences between some patients and the formation of smaller clusters may be due to the different age of the neutrophils measured, but also to the age or gender of the patients or to a minor change in the pre‐analytical conditions. The overall level of biological variability among the selected peaks ranged from 19% to 27%, which is the normal expected value for biological samples.

The richness of Raman spectra highlights the potential of this technique as a valuable research tool for investigating the internal biochemical profiles of neutrophils. Our goal was to use RS to develop a single, easy‐to‐use procedure to describe the molecular properties of neutrophils under physiological conditions. The identification of stable Raman peaks associated with proteins, nucleic acids, and carotenoids further strengthens the value of this method for future applications. These molecular features could potentially serve as a reference for detecting functional changes during immune responses, activation, or disease progression. The most prominent signals were associated with carotenoids. The intensity of these signals is likely to be affected by pre‐resonance conditions, where the absorption bands of the carotenoids align with the 532 nm laser excitation.

An important aspect of this study is the use of 532 nm excitation, which provides pre‐resonance enhancement for intracellular chromophores such as carotenoids. Although near‐infrared excitation is commonly applied in biomedical Raman studies to minimize autofluorescence, visible excitation offers a clear advantage for the sensitive detection of carotenoid‐associated Raman bands due to their strong electronic absorption in the visible range. This excitation wavelength, therefore, contributes to the high signal‐to‐noise ratio and reproducibility of the recorded neutrophil spectra.

Our study has some limitations. Variability in the spectra may also be attributed to the age of the neutrophils, ranging from younger to older cells. While we confirmed the absence of acute illness in donors, it is possible that other undetected conditions or the early stages of the disease could have subtly affected the cells. Additionally, artifacts like contributions from staining agents and other chemicals used during sample processing could affect the spectra. The foundation for incorporating Raman‐based spectral cytometry into current hematological workflows is laid by this protocol. This approach enables future studies to investigate functional or pathological deviations using a standardized reference by recording stable spectral signatures of neutrophils at rest. This approach might be a useful addition to traditional cytometry methods because it works well with blood smear preparations.

## 5 Conclusion

In this study, we used RS to develop a protocol for obtaining, processing, and analyzing neutrophils. Our band assignments show that it is possible to use Raman microscopy to investigate the molecular fingerprint of neutrophils in their physiological state. The strongest signal of carotenoids was found in neutrophils at 388, 471, 637, 706, 1169, 1307, 1348, 1410, 1500, and 1618 cm^−1^. The results show that the spectral parameters have low interindividual variability and high reproducibility. Raman spectroscopy is ideally suited for future automation in cytometric workflows and exhibits great promise for the precise classification of normal neutrophils. Furthermore, other blood cell types, such as those linked to pathological conditions, could be studied using this methodology.

## Author Contributions

**Lenka Vaňková:** conceptualization, data curation, investigation, methodology, writing – original draft, visualization. **Jiří Bufka:** conceptualization, data curation, investigation, methodology, writing – original draft, visualization. **Pavla Šigutová:** writing – review and editing, data curation. **Monika Holubová:** methodology, writing – review and editing, supervision. **Věra Křížková:** conceptualization, writing – review and editing, supervision.

## Funding

This work was supported by Ministerstvo Školství, Mládeže a Tělovýchovy (Cooperatio MED/DIAG), Univerzita Karlova v Praze (SVV 2026 260 773).

## Ethics Statement

The study protocol was reviewed and approved by the Ethics Committee of the Faculty of Medicine and University Hospital in Pilsen, Charles University, under approval number 458/22. Written informed consent for the use of blood samples in research was obtained from all participants prior to blood donation.

## Conflicts of Interest

The authors declare no conflicts of interest.

## Data Availability

The data that support the findings of this study are available from the corresponding author upon reasonable request.
